# Patient-Perceived Quality of Life after Total Hip Arthroplasty: Elective versus Traumatological Surgery

**DOI:** 10.5402/2011/910392

**Published:** 2011-07-05

**Authors:** Alessandro Aprato, Alessandro Massè, Francesco Caranzano, Renato Matteotti, Patrick Pautasso, Walter Daghino, Michael Kain, Giorgio Governale

**Affiliations:** ^1^Orthopaedic and Traumatological Department, S. Luigi Gonzaga Hospital of Orbassano, University of Turin, 72 Corso Lanza, Torino, 10131 Turin, Italy; ^2^Orthopaedic Department, Mauriziano Umberto I Hospital of Turin, University of Turin, 1 Via Magellano, 10128 Turin, Italy; ^3^Pelvic Surgery Department, Orthopaedic and Traumatological Hospital of Turin, University of Turin, 10 Regione Gonzole, Orbassano, 10043 Turin, Italy; ^4^Department of Radiology, Orthopaedic and Traumatological Hospital of Turin, University of Turin, 33 via Zuretti, 10100 Turin, Italy; ^5^Boston Medical Center, 850 Harrison Avenue, Boston, MA 02118, USA

## Abstract

*Purpose*. The aim was to evaluate and compare patient's health-related quality of life after THA for osteoarthritis and femoral neck fracture. The postoperative outcome was retrospectively evaluated in patients who underwent THA with an intracapsular femoral neck fracture (Group A) or with an hip osteoarthritis (Group B). *Methods*. Length discrepancy was measured on postoperative X-rays. Study groups were compared as to age, results of WOMAC and SF-36 tests, limb length discrepancy (LLD) by independent group *t*-test. Correlations between LLD and results obtained atWOMAC test were performed. 117 patients were enrolled. The 2 groups were similar as to age, type of implanted stem and sex. Mean follow up was 2,4 years for group A and 2,3 years for group B. *Results*. WOMAC score was found higher in group A in all items examinated. Correlation tests did not indicate a statistically significant linear relationship between LLD and WOMAC score in both groups. *Conclusions*. Patients who received THA for arthritis have better perception of quality of life than traumatologic patients. Although LLD should always be strongly considered by the surgeons performing a THA, LLD alone can't be considered as an indicator of patient dissatisfaction or clinical bad result after a 2-year followup.

## 1. Introduction

Total hip arthroplasty is a common treatment for osteoarthritis of the hip or for intracapsular femoral neck fractures. With over 30 years of follow-up data, many studies [1–5] have shown that total hip arthroplasty is an effective treatment for both conditions. For this reason, total hip replacement (THA) has proven to be a reliable procedure and one author has defined it as the “the operation of the century” [6]. In USA, 150,000 to 200,000 hip prostheses are implanted each year and over a million worldwide. As the population continues to live longer, allowing for increased activity level for longer, there is an expectation for the prothesis to functionally last longer and be more durable [7, 8]. Although, THA is considered a successful operation, the underlying condition requiring the patient to undergo THA may affect outcome.

Patients undergoing THA for traumatic conditions have been reported to have lower functional outcome scores [9]. When total hip arthroplasty is performed for a traumatic condition such as an acute femoral neck fracture rather than for a degenerative condition (i.e., primary osteoarthritis, congenital hip displasia) the restoring biomechanics of the hip with the THA are perceived differently. In a traumatic condition, patients go from having a normal hip to a prosthetic hip, where as in the degenerative condition, the patient learns to compensate for the anatomic changes over time and usually is living with pain for some time. Restoring the hip's biomechanics seems to provide more relief in the degenerative condition. One reason for this, may be related to the patients' perception of any change in leg length. In the traumatic condition anatomic landmarks are distorted and restoring normal lengthen maybe more difficult. 

In this prospective study, we sought to evaluate and compare the clinical outcomes and patients health-related quality of life (HRQoL) after THA for either osteoarthritis or femoral neck fracture. Additionally, we aimed to evaluate if there were any differences in leg length between the two groups, and whether or not this affected the clinical outcome of patients.

## 2. Materials and Methods

Radiographical controls and postoperative outcome were prospectively collected in patients who underwent THA between March, 2002 and May, 2005 in the orthopaedic clinic of Turin's university. Patients presenting with an intracapsular femoral neck fracture (Group A) or with a diagnosis of primary osteoarthritis (OA) (Group B) requiring primary THA were included in the study. OA was defined by the American College of Rheumatology's clinical classification criteria for OA of the hip [10].

Radiographical appearance of good bone quality suggesting the use of uncemented components was a specific inclusion criteria.

Patients were excluded for the following reasons: age over 75 or under 55, previous surgery to the index joint, inflammatory arthritis, relevant comorbidity (tumors, diabetes mellitus, history of respiratory disease, cardiovascular disease, mental health problems, musculoskeletal system diseases, previous endocrinological diagnosis) or refusal to answer to the questionnaires. 

Patients with one or more of relevant comorbility were excluded because general diseases could affect health-related quality of life.

Every patient gave his written consent to participate to this study. All patients were operated by the same group of 5 surgeons by posterolateral approach. Uncemented Zweymüller Alloclassic stems, with a 28 mm metall heads and the FITMORE acetabular components with ultra-high-molecular-weight polyethylene inserts (Zimmer, Warsaw, Ind), were implanted in all patients enrolled.

Patients followed the same rehabilitation protocol with crutches assisted weight-bearing starting from the second day postoperatively and full weight bearing after 40 days. 

Clinical, radiographical data were collected postoperatively and at the 2-year follow-up visit. Patients lost to followup were called, and a new appointment was scheduled. The clinical evaluation was performed at 2 years from the prosthetic implant to insure a maximum recovery from surgery [11]. 

Every patient completed the Western Ontario McMaster (WOMAC) Osteoarthritis Index [12, 13] and Medical Outcomes Study Short Form 36 (SF-36) [14] questionnaire.

WOMAC includes dimensions for pain (5 items), stiffness (2 items), and function (17 items). Dimensions are equally weighted and reported as sums, where the higher number indicates a greater burden of OA. The WOMAC questionnaire contains 24 questions, each question is given a Likert scale response from 0 (best health state) to 4 (worst health state). The score for each subscale is calculated as the sum of the scores of each question included in the subscale. The range of each subscale is ranged as follows, function, 0–68; pain, 0–20; stiffness, 0–8 points. 

The SF-36 is a validated outcomes survey currently used to measure the public health of populations as well as to compare the health of patients with different medical conditions. The SF-36 measures three major health attributes (functional status, well being and overall health) in eight subscales. These are (1) general health, (2) physical functioning, (3) role limitations due to physical health, (4) role limitations due to emotional problems, (5) social functioning, (6) pain, (7) energy/fatigue, and (8) emotional well being. The SF-36 scores range on a scale of 0–100 (from worst to best). The eight subscales provide a health profile. The SF-36 has been translated and validated for the Italian language.

Changes in leg length related to the hip replacement was measured on the digital radiographs with a dedicated software (Synchromed, Fuji) in anteroposterior pre and post-operative views: using a line connecting the lowest part of the ischial tuberosities, the intersection of the line on both femurs will be gauged from the highest part of the lesser trochanters to measure the leg length inequality. 

All the patients gave the informed consent prior being included into the study; the study was authorized by the local ethical committee and was performed in accordance with the Ethical standards of the 1964 Declaration of Helsinki as revised in 2000. 

### 2.1. Statistical Analysis

Data were recorded in a custom made database and analysed by a commercial software package (TexaSoft, WINKS SDA Software, 6th Edition, Cedar Hill, Texas, 2007). 

The study groups were compared as to age, sex, results of WOMAC and SF-36 tests, limb length discrecancy by independent group *t*-test.

Correlation between changes in leg length related to the hip replacement and results obtained at WOMAC test were performed using Pearson's correlation coefficient.

Statistical significance was stated at *P* < 0.05 for all the tests performed.

## 3. Results

There were 523 THA performed between March, 2002 and May, 2005 with 117 eligible for inclusion to the study: 52 in group A (37 females and 15 males) and 65 in group B (43 females and 22 males). The mean age was 66.4 years in group B and 69.3 years in group A. Mean follow up was 2.4 years for group A and 2.3 years for group B with no statistically significant difference (*P* = 0.08). There was no statistical difference between the two groups with regard to gender (*P* = 0.076), age (*P* = 0.065), or followup (*P* = 0.12). Both groups underwent the same postoperative physical therapy course. 

**Figure 1:**
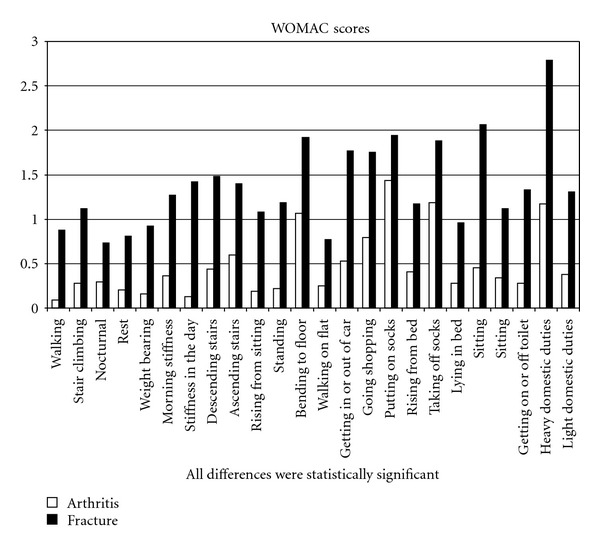
Single item's results at Womac test.

**Figure 2:**
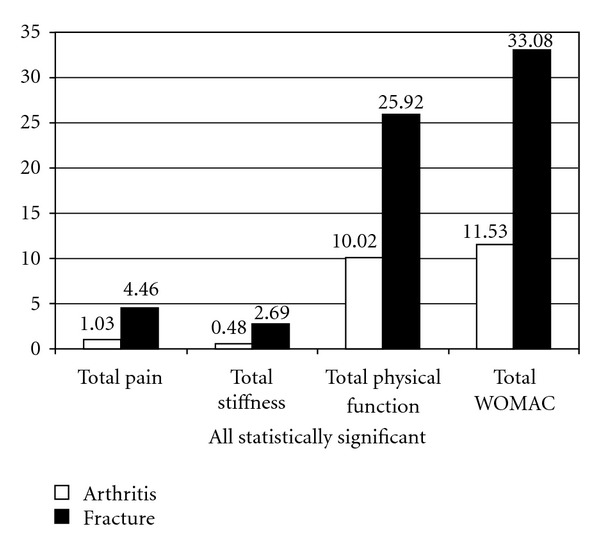
Womac score: dimension's results.

In evaluating the clinical scores, the traumatologic patients had statistically worse results for all items of the WOMAC (*P* = 0.03) (Figure 1). The mean global WOMAC score was 33.1 and 11.5 points for group A and B, respectively. As for the individual components of the WOMAC, the mean “pain” score was 1.03 points for group B and 4.46 for group A, the mean score for “stiffness” was 0.48 (B) and 2.69 (B)points and the mean score for “physical function” was 10.01 (B) and 25.02 (A) points as shown in Figure 2.

**Figure 3:**
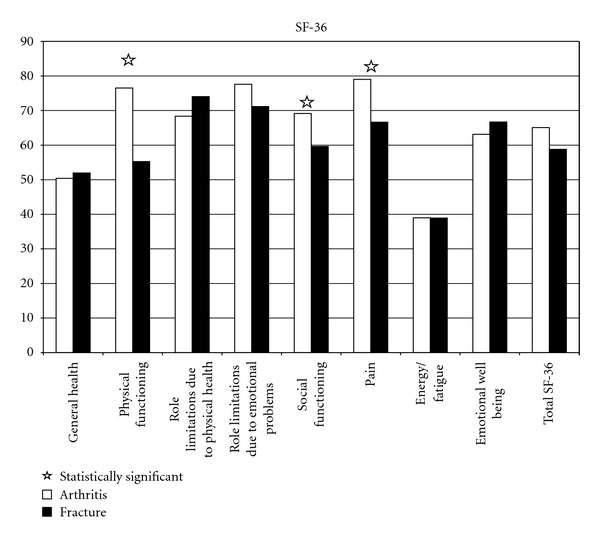
Results at SF-36 test.

SF-36 test scores are shown in Figure 3. Elective patients also obtained statistically better results in these 3 subscales (physical functioning, social functioning and pain) and in the 11 questions (vigorous activities, moderate activities, lifting groceries, climbing several stairs, bending, walking mile, bathing, bodily pain, calm, tired, worse health). Fractured patients did obtained statistically better results (*P* = 0.03) than the arthritis patients in general health and the limitation due to physical health. The fracture group also had improvement in the emotional well-being subscale, but this improvement was not statistically different (*P* = 0.08). All the others subscales were higher for elective patients.

Regarding leg length, the mean postoperative changes following hip replacement on postoperative X-rays were 0.18 cm of shortening for elective group and an overlengthening of 0.52 cm for fractured group. This difference was not statistically significant (*P* = 0.64) between the two groups, and no correlation was found between CLL and WOMAC score in both groups (*P* = 0.43).

## 4. Discussions

The goal of our study was to compare the outcomes of patients undergoing THA for osteoarthritis to those undergoing THA for femoral neck fractures, using the WOMAC index and the SF-36 test. The results obtained at Womac and SF-36 were similar to those reported in the literature [14, 15] for elective and traumatologic patients, but in our study, the HRQoL of traumatologic patients was significantly worse than in patients with osteoarthritis. 

These results are in contrast with the common surgical finding of retracted and reduced muscular and tendon function in patients with chronic disease such as osteoarthritis. As previously reported [16, 17], patient expectations may play an important role in determining the outcome of THA. Analysing the SF-36, we reported similar results between the two groups in general health and emotional well being, but physical functioning was significantly different; limitations were due to physical problems for elective patients while emotional problems caused the major limitations for traumatologic patients. It appears from our data that the traumatologic patients have higher expectations than elective ones. For example, a 57-year-old golfer who remains unable to complete 18 holes after a traumatic primary hip replacement might well regard the operation as a failure despite a hip score that would categorise him as good or excellent. Conversely, a 63-year-old arthritis patient confined to chair whose surgery has restored domestic independence, with commensurate improvement in quality of life. Patient's satisfaction can, therefore, be poor if expectations are not met. In conclusion, it is important to understand the differences in treating degenerative patients versus traumatic patients, and this study provides some insight into how hip fracture patients perform functionally after THA. This information will help educated patients and allowed for a more detailed informed consent.

Changes in leg length related to the hip replacement are common after hip arthroplasty. In traumatologic patient the preoperative planning is usually performed on the opposite healthy side; furthermore the absence of anatomical landmarks in fractured hips could lead the surgeon to a less accurate restoration of the CLL. Different studies [18–20] showed a correlation between CLL after THA and back pain and sciatica, gait disorders, general dissatisfaction, and dislocation. On the contrary one paper [21] in the orthopaedic literature suggests that CLL has no effect on the functional outcome of THA while most surgeons [22–24] believe it is important to address and employ various methods of templating and of intraoperative methods to evaluate leg length. In fact, when shortening exceeds 10 mm and lengthening exceeds 6 mm, patients can perceive the difference and that these extremes CLL can influence the clinical result [25]. 

In our study, in both groups there, was no difference in leg length, and this lead us to exclude limb over lengthening (within the range measured) as cause of the different quality of life perceived by fractured patients. However, our study can not confirm that CLL (in the range described in literature) after THA is associated with patient dissatisfaction; in fact, there was no correlation between CLL and the clinical results. This conclusion should be evaluated according to the number of patients enrolled and to the mean measured CLL (in both groups inferior to 1 cm); therefore, small changes in leg length do not appear to affect patient satisfaction between these two groups. The limitation of this conclusion is that leg length inequality measurements on an AP pelvis are not always adequate and true limb length discrepancy was not measured so preexisting inequalities outside the imaged area might have contributed to ongoing difficulties. 

Despite the limitations of a low number of patients enrolled and the method of measuring leg length, it seems that patients who received THA for arthritis have better perception of quality of life than traumatic hip fracture patients. 

Although leg length should always be strongly controlled for by the surgeons performing a THA, differences in leg length cannot be considered as the cause for these differences between these two groups after a 2-year followup.

##  Conflict of Interests

The authors declare that they have no competing interest. 

##  Authors' Contributions

A. Aprato, A. Biasibetti, and A. Massè conceptualized rationale and design of the study. A. Aprato, R. Matteotti, and F. Caranzano performed the clinical evaluation. P. Pautasso and A. Aprato performed radiologic measurement. A. Aprato e A. Massè performed the statistical analyses. M. Kain performed the english revision of the manuscript. All authors read and approved the manuscript.

## Abbreviations

THA: Total hip arthroplasty
HRQoL: Health related quality of life
WOMAC: Western Ontario McMaster (WOMAC) Osteoarthritis Index
SF-36: Medical Outcomes Study Short Form 36
CLL: Changes in leg length related to the hip replacement.
